# Effects of T6 Treatment, Tensile Temperature, and Mass Fraction of SiC on the Mechanical Properties of SiC_p_/6061Al Composites

**DOI:** 10.3390/ma12101602

**Published:** 2019-05-16

**Authors:** Yong Hu, Tong Wu, Yue Guo, Wenyang Wang, Mingkai Song, Long Qian, Hongwei Zhao, Maosen Wang

**Affiliations:** 1 School of Mechanical and Aerospace Engineering, Jilin University, Changchun 130025, China; huyong@jlu.edu.cn (Y.H.); twu18@mails.jlu.edu.cn (T.W.); yueguo15@mails.jlu.edu.cn (Y.G.); wwy18@mails.jlu.edu.cn (W.W.); mksong18@mails.jlu.edu.cn (M.S.); qianlong17@mails.jlu.edu.cn (L.Q.); hwzhao@jlu.edu.cn (H.Z.); 2 Key Laboratory of CNC Equipment Reliability, Ministry of Education, Jilin University, Changchun 130025, China; 3 College of Construction Engineering, Jilin University, Changchun 130012, China

**Keywords:** SiC_p_/6061Al composites, microstructure, critical reinforcement concentration, relative density, tensile property, fracture morphology

## Abstract

SiC_p_/6061Al composites have been developed and widely applied in many fields, such as automobile, aerospace, shipping, and so on. Considering heat treatment, service environment, and strength of composites, this paper comprehensively studies the mechanical properties of SiC_p_/6061Al composites with a large range of SiC mass fractions under T6 treatments and different tensile temperatures. SiC_p_/6061Al composites were successfully prepared by hot press sintering at various SiC mass fractions (0–30%), and the influences of SiC concentration and T6 treatment on the mechanical properties of composites were characterized via tensile tests at room temperature, 100, and 200 °C. Microstructure and fracture surfaces of composites with various SiC concentrations were further analyzed by optical microscope and SEM. The formula for the biggest critical reinforcement concentration for the saturated distribution of SiC is proposed to reveal the strengthening rule of different SiC concentrations. Results show that the effect of T6 treatment on the mechanical properties of composites is a marked increase in tensile strength and an obvious decrease in elongation. The increase in the SiC mass fraction, except at 30%, is able to bring an increase in tensile strength and a decrease in elongation, and the change of the elongation is insignification in T6-treated specimens. The tensile strength of T6-treated specimens decreases as temperature increases, and the composite has a maximum elongation at 100 °C.

## 1. Introduction

6061 aluminum alloy matrix composites, with broad application prospects in the fields of automobile, aerospace, shipping, etc., have been highlighted recently due to the high thermal conductivity, low density, and good processing performance of the 6061 aluminum alloy. On the other hand, SiC particles are suitable as reinforcement due to their high hardness, high strength, and excellent wear resistance [1,2,3,4,5,6,7]. Many scholars have studied the reinforcement of SiC on aluminum alloy matrix composites, but few papers have studied the mechanical properties of SiC_p_/6061Al composites with a large range of SiC mass fractions, especially connecting this with heat treatment and different tensile temperatures.

Kumbhar et al. prepared composites by changing SiC contents (0, 3, 6, and 9 wt.%) and proposed that improvements in the mechanical properties of the 6061 aluminum alloy matrix can be obtained with the addition of SiC [1]. Prabhu et al. found that the tensile strength of composites increased significantly with increasing SiC content (4 wt.% to 10 wt.%) [8]. Rodríguez-Castro et al. noticed that the mechanical properties of the composites were greatly influenced by the mass fractions of SiC, and that the effect of SiC particles reinforcement on the strengthening of aluminum alloy was limited up to a certain volume fraction [9]. Yan et al. discussed that the tensile strength of the composite decreased when the volume fraction of SiC exceeded a critical value [7]. Therefore, it is important to determine the strengthening rule of different SiC contents for the 6061 aluminum alloy within a large range of SiC mass fractions, and determine the critical concentration for reinforcement.

Since 6061 aluminum alloy is an age-strengthened alloy, it is necessary to consider the effect of heat treatment apart from SiC mass fraction. Zhang et al. concluded that the grain-coarsening rate of the composite was lower than that of the matrix alloy due to the presence of SiC_p_ in the grain boundaries during the solution treatment to improve the tensile strength [3]. Prabhu et al. found that heat treatment had a significant effect on the microhardness of Al6061 matrix alloy and its composites [8].

SiC_p_/6061Al composites are often used at high temperatures, so studying the effect of temperature on the mechanical properties of composites is also important. Shin et al. found that the mechanical properties of composites, such as Young’s modulus, strength, and the strain hardening rate, were markedly increased, and the percentage elongation significantly decreased, with an increase in the volume fraction of reinforcement at room temperature, and that the beneficial effect on strengthening was largely lost at 200 °C [10]. 

Considering heat treatment, service environment, and strength of composites comprehensively, this paper mainly studies the effects of T6 treatment and tensile test temperature on the mechanical properties of SiC_p_/6061Al composites with various mass fractions of SiC particles, which has important guiding significance for the practical application of composites.

## 2. Materials and Methods 

### 2.1. Material Preparation

**Figure 1:**
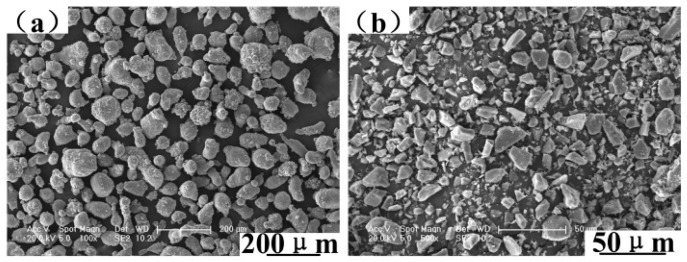
Morphologies of (**a**) 6061 aluminum alloy powder and (**b**) SiC powder.

**Figure 2:**
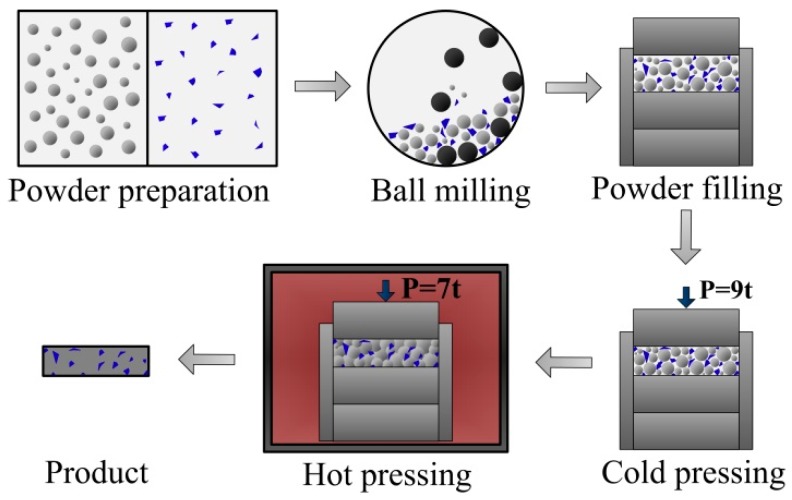
The manufacturing process of composites.

**Figure 3:**
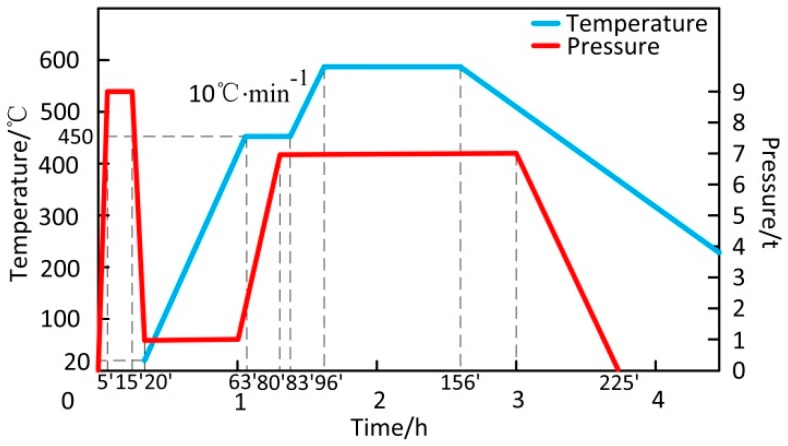
The preparation process and parameters in the vacuum hot pressing sintering furnace.

The experimental material was a 6061 aluminum alloy matrix composite reinforced by various mass fractions of SiC particles. 6061 aluminum alloy powder and SiC powder came from Beijing Xing Rong Yuan Technology Company (Beijing, China), and the purity of SiC powder was greater than 99.5%. The standard chemical composition of the 6061 aluminum alloy is given in Table 1. 6061 aluminum alloy powders are of irregular spherical shape with an average diameter of 48 μm, and SiC particles are of polygonal shape with an average diameter of 10 μm, as shown in Figure 1. SiC particles were added in 0%, 5%, 10%, 15%, 20%, 25%, and 30% by quality, and these composites were marked 0%SiC/6061, 5%SiC/6061, 10%SiC/6061, 15%SiC/6061, 20%SiC/6061, 25%SiC/6061, and 30%SiC/6061, respectively. The manufacturing process of composites is shown in Figure 2. Firstly, the SiC powder and the 6061 aluminum alloy powder, weighed by the electronic balance, were fully mixed in a ball mill for 10 hours, wherein mass fractions of the SiC powder were 0%, 5%, 10%, 15%, 20%, 25%, and 30%, respectively. The homogeneously mixed powders were placed in a graphite mold with an inner diameter of 60 mm, and the graphite mold was placed in a vacuum hot-pressing sintering furnace (Shanghai Chenhua Electric Furnace Corp, Shanghai, China) for cold pressing and hot pressing. The preparation process and preparation parameters in the vacuum hot pressing sintering furnace are shown in Figure 3. In a vacuum environment, mixed powders were cold pressed by 9 t pressure. After the pressure was released, the temperature of the vacuum hot-pressing sintering furnace (Tianjin Zhonghuan Furnace Corp, Tianjin, China) was increased by 10 °C per minute to 450 °C for 20 min to facilitate the discharge of gas between powders, and the pressure reached 7 t at the same time. Subsequently, powders were sintered at 580 °C and 7 t for 60 min. After the temperature had dropped for a while, the pressure was released slowly. Finally, the composite was taken out of the graphite mold to complete the hot-press sintering process.

To verify the effect of heat treatment on the mechanical properties of SiC_p_/6061Al composites, T6 treatment was applied to some SiC_p_/6061Al composites. A vacuum/atmosphere tubular electric furnace was used. SiC_p_/6061Al composites were solution-treated at 530 °C for 30 min under argon protection and then quenched in water. Artificial aging was also carried out under argon protection at 200 °C for 10 hours.

### 2.2. Mechanical Tests

Density test, microstructure observations, and X-ray diffraction (XRD, Rigaku Corporation, Tokyo, Japan) were carried out on un-heat treated composites. Room temperature tensile tests, high-temperature tensile tests, and fracture morphology observations were carried out on both heat-treated and un-heat-treated composites.

Five independent tests were conducted for each composite. The relative density *d* of SiC_p_/6061Al composites was calculated by Equation (1), wherein *ρ*_1_ is the density of SiC_p_/6061Al composites measured by the Archimedes’ principle, and *ρ*_SiC_ and *ρ*_6061_ are the theoretical densities of SiC and 6061 aluminum alloy, respectively (*ρ*_SiC_ = 3.2 g/cm^3^, *ρ*_6061_ = 2.7 g/cm^3^) [11]. *V*_SiC_ and *V*_6061_ are the volume fractions of SiC and 6061 aluminum alloy, respectively, which can be calculated according to Equations (2) and (3) when mass fractions *W*_SiC_ and *W*_6061_ are known:

(1) $$d=\frac{\rho_{1}}{\rho_{\mathrm{SiC}}V_{\mathrm{SiC}}+\rho_{6061}V_{6061}},$$

(2) $$V_{\mathrm{SiC}}=\frac{\rho_{6061}W_{\mathrm{SiC}}}{\rho_{\mathrm{SiC}}W_{6061}+\rho_{6061}W_{\mathrm{SiC}}},\;\mathrm{and}$$

(3) $$V_{\mathrm{SiC}}+V_{6061}=100\%.$$

**Figure 4:**
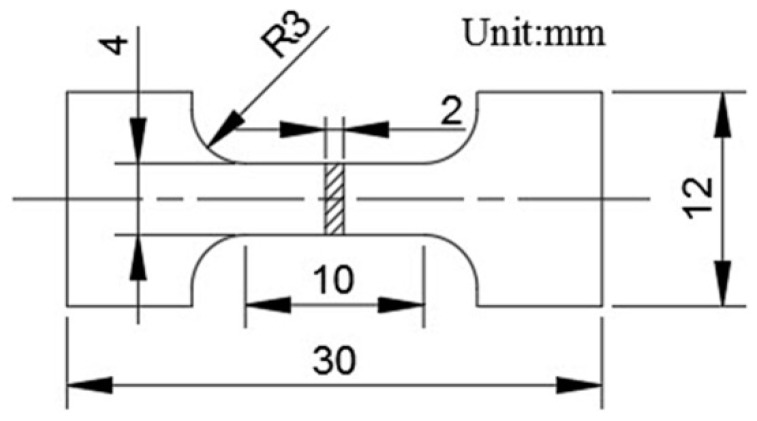
The dimensions of the tensile specimen.

Tensile specimens were cut from the central region of each SiC_p_/6061Al composite. The dimensions of the tensile specimen are shown in Figure 4, with a gauge length of 10 mm, a cross section of 4 mm long, and a width of 2 mm. The tensile test was carried out by using an electronic universal testing machine (Changchun Institute of Applied Chemistry Chinese Academy of Sciences, Changchun, China) with a loading velocity of 0.15 mm·min^−1^. Three independent tests were conducted for each composite. For the high-temperature tensile test, a specimen in a fixture was enclosed in a furnace. The specimen was held at the desired temperature for 30 min before starting the test. The high-temperature tensile tests were conducted at 100 °C and 200 °C.

Metallographic specimens with lengths of 2 mm and diameters of 10 mm were cut from the central region of each SiC_p_/6061Al composite and then ground and polished. The microstructure and distribution of SiC particles in the specimen were observed using optical microscopy (Olympus, Tokyo, Japan). X-ray diffraction (XRD) was used to make the phase identification of composites. In order to verify the detailed fracture mechanism of composites, the fracture surface of the specimen after the tensile test was observed by a scanning electron microscope (Japan Electron Optics Laboratory, Tokyo, Japan).

## 3. Results and Discussion

### 3.1. Microstructure

**Figure 5:**
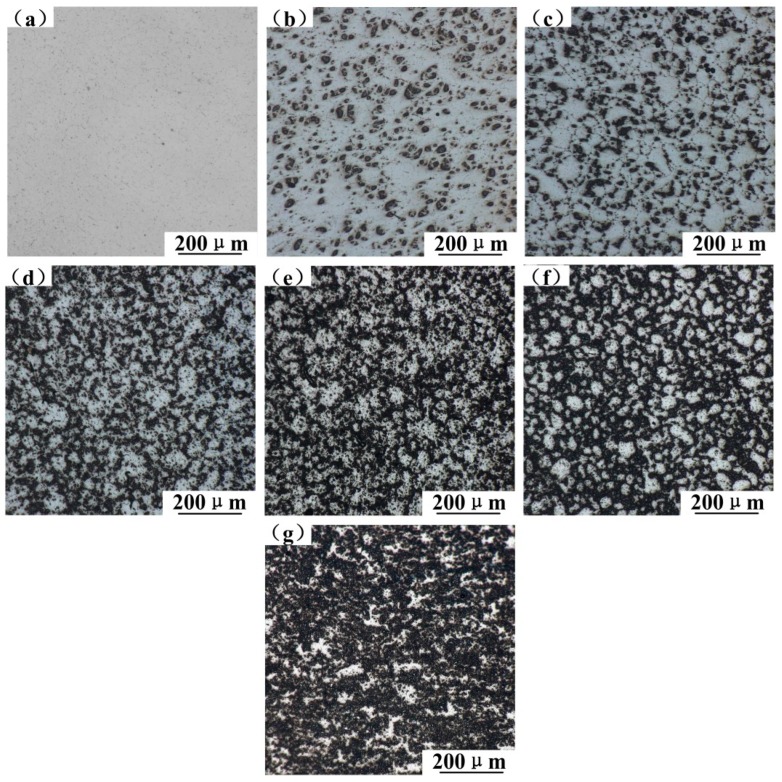
Optical micrographs of SiC_p_/6061Al composites: (**a**) 0%SiC/6061; (**b**) 5%SiC/6061; (**c**) 10%SiC/6061; (**d**) 15%SiC/6061; (**e**) 20%SiC/6061; (**f**) 25%SiC/6061; (**g**) 30%SiC/6061.

To research the effect of T6-treated and tensile temperature on the mechanical properties of SiC_p_/6061Al composites, prepared composites should satisfy the conditions of high relative density, homogeneous particle distribution, and so on. Figure 5 shows the optical micrographs of 200× magnification of SiC_p_/6061Al composites without T6-treated. The SiC_p_/6061Al composites with SiC mass fractions from 0% to 30% had no visible holes in these optical micrographs. Microstructural studies demonstrated that the composites exhibited good compactness, and there were excellent boundaries between the matrix alloy and reinforcing particles. Two phenomena were observed in the distribution of SiC particles in the matrix alloy: firstly, when the SiC mass fraction was less than 10%, SiC particles were homogeneously distributed in the matrix alloy. When the SiC mass fraction reached 10%, SiC particles aggregated to form SiC clusters. Slipenyuk et al. pointed out that, in order to make the reinforcement particles distribute homogeneously in the composites, a theoretical limit for the reinforcement concentration exists [12]. The theoretical limit depends on the particle size ratio of matrix and reinforcement, the particle shape, and the processing method, and a formula has been proposed to calculate the critical reinforcement concentration of volume fraction *V_h_* for homogeneous distribution of SiC:

(4) $$V_{h}=\alpha \frac{V_{r1}}{V_{r1}+V_{m}}=\alpha \cdot \left[1-{\left(1+{\left(\frac{d}{D}\right)}^{3}+3{\left(\frac{d}{D}\right)}^{2}+3\frac{d}{D}\right)}^{-1}\right],$$

where *α* (*α* = 0.18) is a parameter that is considered to take into account the effects of reinforcement connection, the particle shape, the particle size distribution, and the reinforcement of local deviations in the composite. *V_r_*_1_ and *V_m_* are the volume of matrix particles and reinforcing particles in the composite, respectively, and *d*/*D* is the reinforcement-matrix particle size ratio.

Secondly, SiC particles preferentially aggregated at the boundary of Al particles. When the SiC mass fraction reached 25%, as shown in Figure 5f, it was obvious that SiC particles aggregated at the boundary of Al particles. This phenomenon has been proposed in some papers. Gubicza et al. reached a conclusion that the SiC_p_/matrix interfaces belonged to an inherent interface because of the differences between the corresponding crystal structures and lattice constants [13]. Ogel et al. mentioned that the reinforcing particles tended to segregate and cluster at the Al powder particle boundaries [14]. When the SiC mass fraction reached 30%, as shown in Figure 5g, the boundary of Al particles was no longer apparent, which is like the boundary of Al particles has reached saturation.

**Figure 6:**
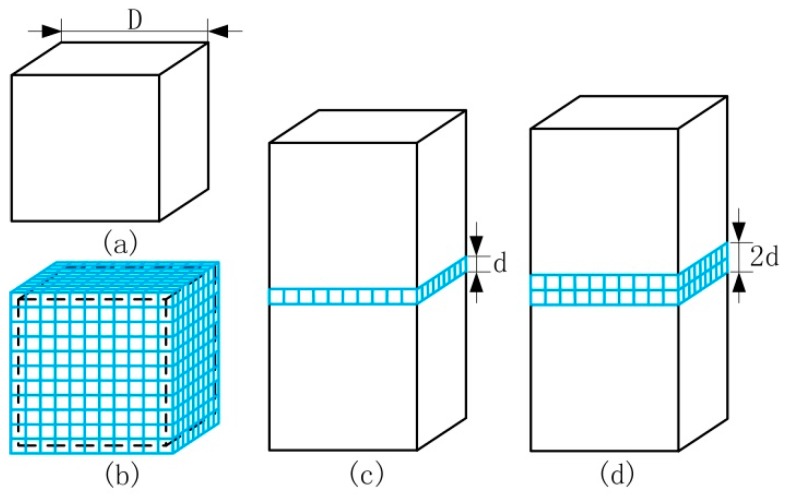
The relative position of reinforcing particles and matrix particles: (**a**) a matrix alloy particle, (**b**) the distribution of reinforcing particles, (**c**) the homogeneous distribution, and (**d**) the saturated distribution.

SiC particles aggregated at the boundary of Al particles, as shown in Figure 6b. When reinforcing particles were homogeneously distributed in the matrix, without the cluster of reinforcing particles, a layer of reinforcing particles was surrounded by two layers of adjacent matrix alloy particles, as shown in Figure 6c. At this time, we calculated the homogeneous distribution of reinforcing particles using the critical reinforcement concentration formula of Equation (4), when *D* = 48 μm and *d* = 10 μm, and the result of the calculation was *V_h_* = 7.80% and the mass fraction *W*_SiC_ = 9.11%. Compared with Figure 5, when the SiC mass fraction was 5%, SiC particles were homogeneously distributed in the matrix alloy. When the SiC mass fraction reached 10%, SiC particles aggregated to form SiC clusters. *W*_SiC_ = 9.11% is a theoretical limit for the reinforcement particles distributing homogeneously in the composites.

For the second phenomena, the critical reinforcement concentration formula for calculating the saturated distribution of reinforcing particles was proposed in this paper and the following assumptions were set. The ball mill used to mix powers homogeneously did not affect the distribution of particles. Cubic reinforcement particles, with the cube side *d* and cubic matrix alloy particles with the cube side *D*, were considered. The shape and size of reinforcing particles after hot-pressing sintering did not change. In this case, the volume *V_m_* of a matrix alloy particle, as shown in Figure 6a, is:

(5) $$V_{m}=D^{3}.$$

When reinforcing particles reached saturation with clusters of reinforcing particles, two layers of reinforcing particles were surrounded by two layers of adjacent matrix alloy particles, as shown in Figure 6d. At this time, the volume *V*_r2_ of reinforcing particles contained around one matrix alloy particle is:

(6) $$V_{r2}={\left(D+2d\right)}^{3}-D^{3}.$$

Therefore, the critical reinforcement concentration recorded as the volume fraction *V*_S_ of the saturated distribution of reinforcing particles is:

(7) $$V_{s}=\beta \frac{V_{r2}}{V_{r2}+V_{m}}=\beta \cdot \left\{1-{\left[1+8{\left(\frac{d}{D}\right)}^{3}+12{\left(\frac{d}{D}\right)}^{2}+6\left(\frac{d}{D}\right)\right]}^{-1}\right\},$$

where *β* (*β* < 1) is a parameter that is considered to take into account the effects of the particle shape, the particle size distribution, and the reinforcement local deviations in the material. In order to obtain the value of *β*, the critical reinforcement concentration of the saturated distribution *W*_SiC_ = 30% (*V*_S_ = 26.56%) in this paper was substituted into Equation (7) and *β =* 0.41.

To verify the obtained formulas, we calculated the composite prepared by Yan et al. when *D* = 45 μm and *d* = 15 μm [7]. In this case, the critical reinforcement concentration of the saturation distribution calculated by Equation (7) was *V_S_* = 32.08%. Compared with Yan et al., when the SiC volume fraction was 30%, the composites achieved the maximum tensile strength [7]. When the SiC volume fraction reached 35%, the tensile strength of the composites decreased. *V*_SiC_ = 32.08% was the biggest critical reinforcement concentration of the saturation distribution, and the strength of the composites had begun to decrease.

**Figure 7:**
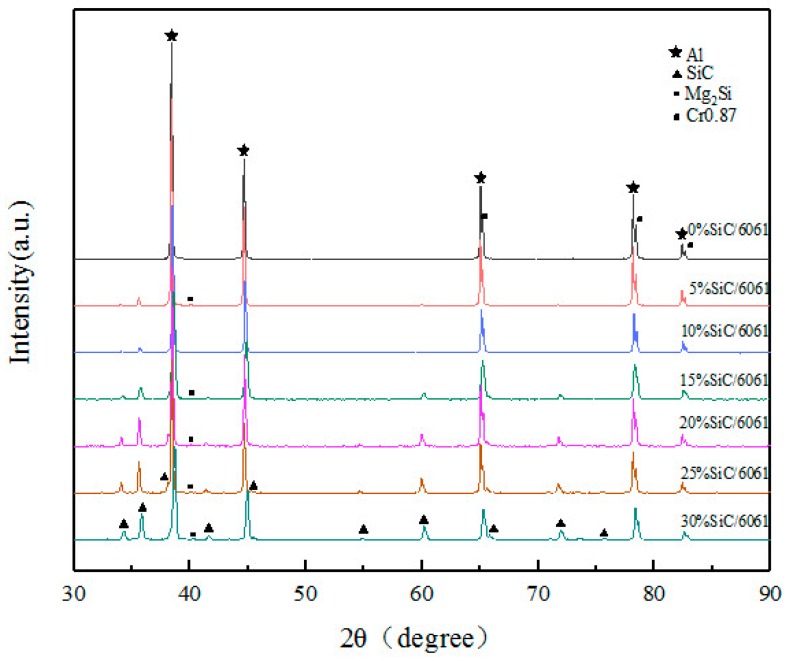
X-ray diffraction (XRD) pattern of SiC_p_/6061Al composites.

The XRD patterns of composites in Figure 5 are shown in Figure 7 to make the phase identification. It was found that the diffraction peaks of SiC became clear with the increase in SiC content. Mg_2_Si is the main eutectic phase for the 6061 aluminum alloy, and Mg_2_Si is a hard and brittle intermetallic phase, which exists in the form of particles distributed along grain boundaries and is easy to crack in tensile tests. The diffraction peak of Mg_2_Si was found in XRD patterns, as shown in Figure 7. Zhang et al. proved that solution treatment can lead to the dissolution of the Mg_2_Si phase into the Al phase, which can improve the strength of composites and reduce the number of crack initiation sites [3]. Because Mg_2_Si exists in a small amount and plays a similar role to SiC particles, the main factors affecting the mechanical properties of composites are the aluminum alloy matrix and SiC-reinforced particles.

### 3.2. Density and Relative Density

**Figure 8:**
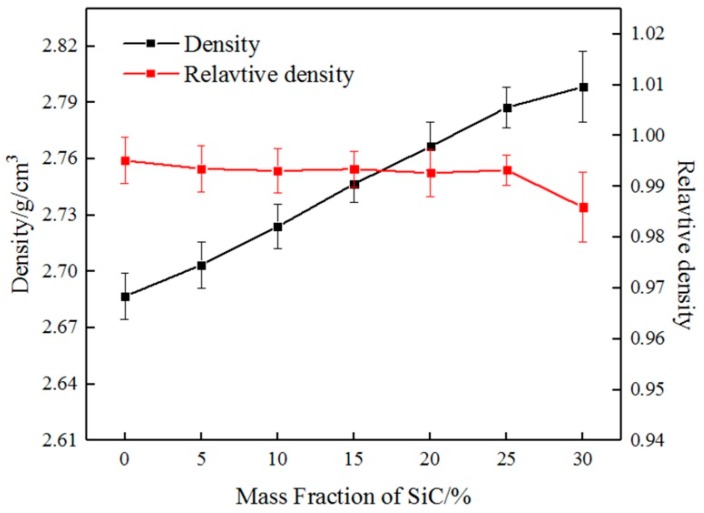
The variations in density and relative density with the mass fraction of SiC.

Using the rule of mixture, the density of the composite should increase with the increasing mass fraction of SiC, where *ρ*_SiC_ = 3.2 g/cm^3^ and *ρ*_6061_ = 2.7 g/cm^3^. The variations in density with SiC content of composites without T6-treated are indicated in Figure 8. It can be seen that the density improved with the addition of SiC.

The variations of relative density with SiC content are indicated in Figure 8. Using the preparation process, high relative density was achieved, which was maintained at about 99% and composites had no visible holes in optical micrographs. The homogeneous distribution of SiC particles in the 6061Al matrix contributed to the high relative density, and the preparation process also helped composites to obtain the nearly full relative density within suitable temperatures and pressures. In addition, the relative density of SiC_p_/6061Al composites decreased with the increase of SiC. The reasons for this follow.

SiC is a ceramic phase with a high melting point and a particularly high stiffness, which is difficult to deform. As the mass fraction of SiC increased, the mass fraction of 6061Al decreased, and the contact area of SiC-SiC increased. When the pores of the contact surfaces of SiC-SiC increased and the plastic deformation of 6061Al could not fill the pores, the relative density reduced. The increase of SiC content led to the decline in the pressing capacity of composites because of the higher hardness of SiC. El-Kady et al. considered that the lower relative density is due to the lower compressibility [15]. In addition, SiC particles are homogeneously distributed to form a dense network to prevent densification of composites [16].

When the mass fraction of SiC reached 30%, the relative density decreased remarkably. Part of the reason is that the higher hardness of SiC leads to lower compressibility of the composite and a lower relative density. *W*_SiC_ = 30% was the largest critical reinforcement concentration of the saturation distribution of SiC particles. When the SiC mass fraction reached 30%, a large amount of aggregation of SiC particles occurred. There were a lot of contact areas of SiC-SiC to reduce the relative density. The SiC network is denser and more cluttered to prevent densification of SiC_p_/6061Al composites. It can be seen that the mass fraction of SiC and the distribution of SiC particles have a great influence on the relative density of composites.

### 3.3. Tensile Properties

**Figure 9:**
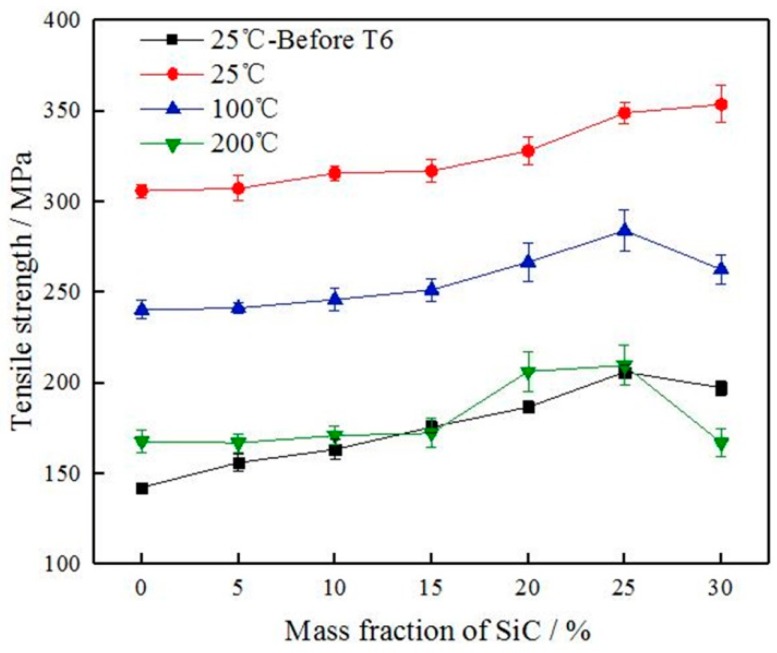
The effect of T6 treatment and test temperature on the tensile strength of SiC_p_/6061Al composites.

**Figure 10:**
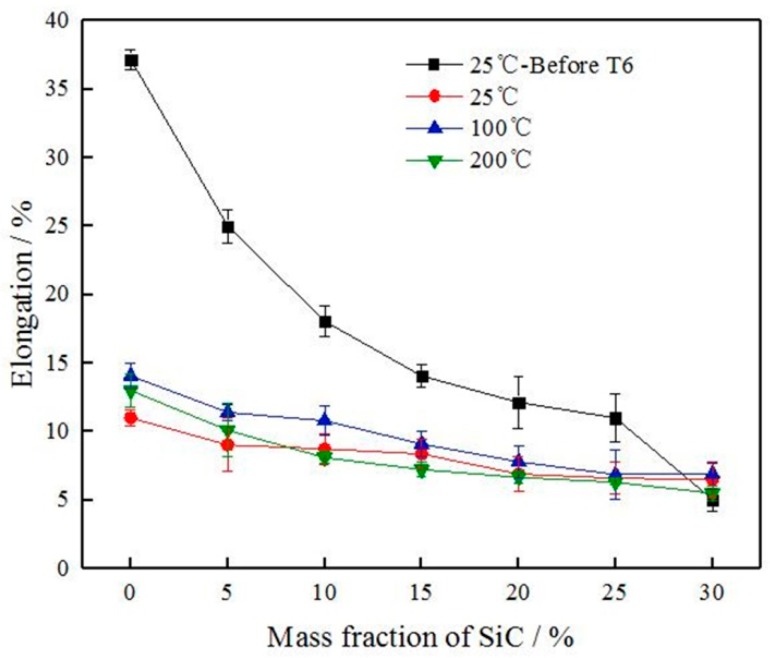
The effect of T6 treatment and test temperature on the elongation of SiC_p_/6061Al composites.

The effect of T6-treated and test temperature on the tensile strength and the elongation of SiC_p_/6061Al composites are shown in Figure 9 and Figure 10.

Experimental results for T6-treated at room temperature indicated that the effect of T6-treated on the mechanical properties of composites caused a marked increase in tensile strength, but a significant decrease in elongation. That is, the T6-treated can significantly enhance the tensile strength, but sacrifices the ductility. For example, the tensile strength of 0%SiC/6061 with T6-treated was increased by 115%, and the elongation was reduced by 70%. The reason for the phenomenon is that the matrix of composites in the 6061 aluminum alloy belongs to an age-hardening alloy. The supersaturated solid solution can be formed in dense composites after solution treatment, and fine precipitates can be dispersed from composites after aging treatment. These fine precipitates can be regarded as barriers to dislocations that reduce the mobility of dislocations and improve the plastic deformation resistance, so the strength of composites increases and the plasticity reduces.

With the increase of SiC particles, the tensile strength of the composite increased and the elongation decreased. The elongation of the composite without T6-treated was very sensitive to the increase of SiC particles, and the elongation decreased went from a sharp decrease to a slow decrease as SiC particles increased. The elongation of the T6-treated composite was not sensitive to the increase of SiC particles, and the elongation decreased insignificantly. The strengthening mechanisms, which may operate in SiC_p_/6061Al composites, can be divided into two categories: direct and indirect strengthening [17]. Direct strengthening is a model based on the load sharing between the reinforcements and the matrix. Since the strength and the ability to withstand stress of the SiC particles are stronger than 6061 aluminum alloy, the matrix is strengthened when SiC particles are added. External stress is generally transmitted from the matrix to the reinforcement through the interface. The number of interfaces increases with the increase in SiC particles, which improves the ability to withstand stress and the tensile strength. Indirect strengthening is a model caused by the changes in the matrix microstructure due to the introduction of reinforcements, and the most common reinforcement mechanisms are coefficient of thermal expansion (CTE) mismatch, grain refinement, and Orowan strengthening [18,19]. The CTE mismatch mechanism is due to the large difference in the thermal expansion coefficients of the 6061 aluminum alloy and the SiC, and the thermal mismatch that results is because the interface is inconsistent in heat expansion and cooling shrinkage [20]. Then thermal residual stress and a large number of dislocations are generated in the 6061 aluminum alloy around the SiC particles, and the strength of 6061 aluminum alloy is improved. The grain refinement mechanism means that the addition of SiC particles during the preparation of composites can refine the grains of 6061 aluminum alloy matrix, so that the strength of composites is improved. Orowan strengthening mechanism points out that the moving resistance of dislocation will increase when the moving dislocations meet SiC particles due to the high strength of SiC particles, belonging to the non-deformable particles, and the dislocation line will bend as it comes across the SiC particles. When the applied load is large enough, the dislocation will continue through the particles and move forward leaving a new dislocation necklace. The SiC particles not only hinder the movement of the dislocation but also generate the dislocation multiplication. Thus, the tensile strength of the composites is improved. Not only did the elongation of composites continue to decrease when the mass fraction of SiC particles reached 30%, but also the tensile strength tended to decrease. SiC particle clusters can form more SiC-SiC interfaces because of the saturated distribution, and the region of SiC particle clusters generally had defects such as holes and impurities. When the composite is subjected to an applied load, these defects are liable to cause a high-stress concentration to become a crack source and cause the generation and expansion of cracks. Therefore, the tensile strength of composites is greatly affected by the mass fraction and the distribution of SiC particles.

The tensile strength of SiC_p_/6061Al composites decreases as the testing temperature increasing, as shown in Figure 9. Atrian et al. pointed out that the properties of SiC particles did not change significantly during the temperature increase, while the 6061 aluminum alloy matrix softened and the dislocations mobility increased with the increasing temperature to reduce the tensile strength of composites [21]. The phenomenon was also similar to that obtained by Li et al. with SiC_p_/2024Al composites [22]. For direct strengthening, external stress is generally transmitted from the matrix to the reinforcement through the interface. Li et al. reached this conclusion through a calculation that showed that high temperature degraded the load-transfer strengthening [22]. For indirect strengthening, dislocation strengthening is significantly dependent on the density of dislocations. The dislocation density decreases with the increasing temperature, and decreasing of the dislocation density weakens the strength of the composites. In addition, the void nucleation in the matrix will be more extensive when the temperature increases, and cracks preferentially initiate in and propagate along with the matrix. These lead to the premature fracturing of the composite before the load-carrying capacity of SiC_p_ was operated completely, which can be demonstrated by the fracture morphologies shown in Section 3.4. Nageswara Rao et al. found that 6061 aluminum alloy is thermally stable up to a temperature of 250 °C with slight grain coarsening [23]. Abnormal grain growth was observed after annealing at high temperatures (300 °C). The effect of grain coarsening on mechanical properties can be disregarded at 200 °C. From the above standpoints, the variations in mechanical properties with the testing temperature can be well interpreted. The elongation of the T6-treated SiC_p_/6061Al composites is not significant in relation to the change of temperature, as shown in Figure 10. The elongation of composites at 100 °C is slightly higher than the elongation of composites at room temperature with T6-treated, but much lower than the elongation of composites without T6-treated. The tensile strength of T6-treated specimens at 200 °C is degraded continuously, and the decrease in elongation is also not obvious at the same time. These phenomena of elongation are explained in detail in Section 3.4 by fracture morphologies.

It can be seen from the discussion that the elongation of T6-treated composites changes insignificantly with the increase of SiC content and tensile temperature, indicating that T6-treated has the greatest influence on the elongation of SiC_p_/6061Al composites. The 6061 aluminum alloy matrix is an age-strengthened alloy, and fine precipitates dispersed after the aging treatment can improve the plastic deformation resistance of composites. In general, heat treatment must be performed before using SiC_p_/6061Al composites. The use of T6-treated SiC_p_/6061Al composites needs to be concerned with the strength in the service environment rather than the elongation.

### 3.4. Fracture Morphology

The fracture morphology is divided into the ductile fracture and brittle fracture according to the amount of fracture plastic deformation. Ductile fracture is characterized by a honeycomb shape in the fracture morphology and the fracture surface consists of dimples under scanning electron microscopy. Brittle fracture is characterized by a relatively flat fracture and there will be more fracture planes on the fracture surface.

**Figure 11:**
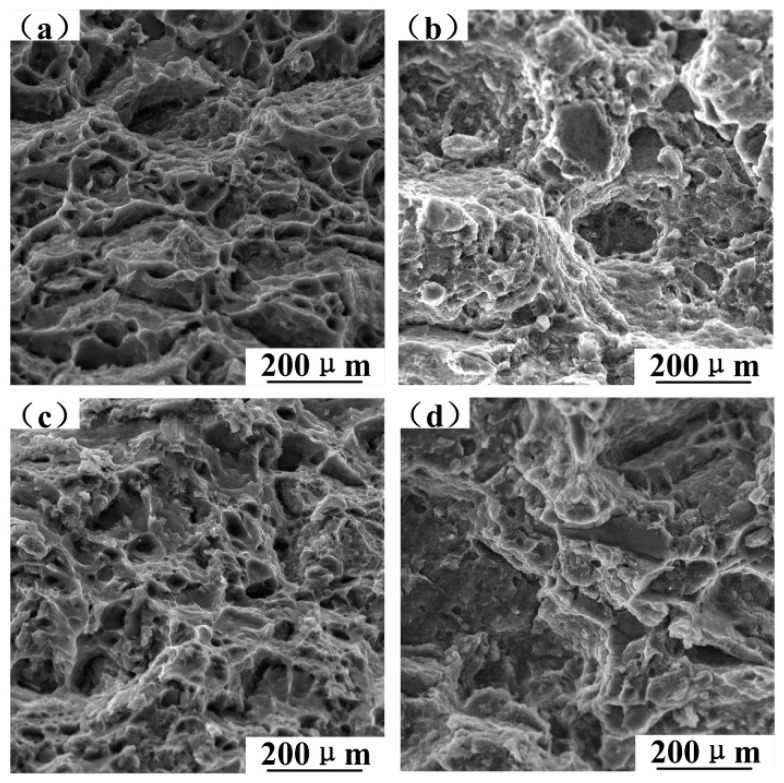
The fracture morphology of T6-treated composites after room temperature tensile test: (**a**) 10%SiC/6061 without T6 treatment, (**b**) T6-treated 10%SiC/6061, (**c**) 15%SiC/6061 without T6 treatment, and (**d**) T6-treated 15%SiC/6061.

Figure 11 shows the fracture morphology of 10%SiC/6061 and 15%SiC/6061 after the room temperature tensile test. No clean separation at the SiC/matrix interface indicates that the interfacial bonding is good. The fracture morphologies of composites without T6-treated, as seen in Figure 11a,c, have a large number of dimples, indicating good plasticity. Only a small amount of dimples are observed in fracture morphologies of T6-treated composites in Figure 11b,d. The plastic deformation resistance of the T6-treated composite is significantly improved, and the fracture of composites changes from ductile fracture to brittle fracture.

**Figure 12:**
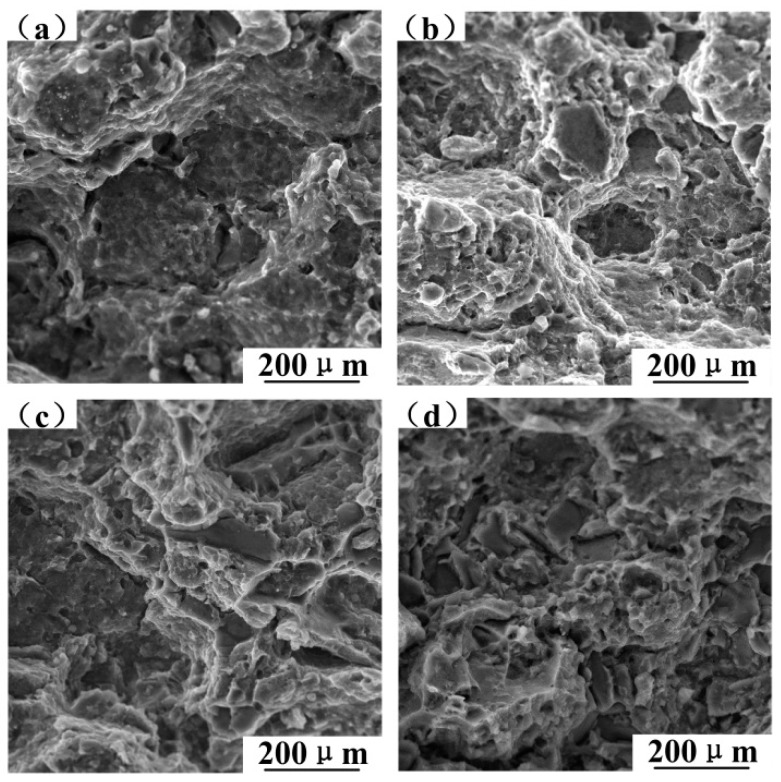
The fracture morphology in relation to SiC content after the room temperature tensile test: (**a**) T6-treated 5%SiC/6061, (**b**) T6-treated 10%SiC/6061, (**c**) T6-treated 15%SiC/6061, and (**d**) T6-treated 20%SiC/6061.

The continuous reduction of dimples and the continuous increment of fracture planes with the increase of SiC content, as shown in Figure 12, result in a reduction in the plastic deformation of composites. SiC is a non-deformable particle with high stiffness which can hinder the deformation of the surrounding matrix and creates high-stress concentrations at the interface. Since the tensile strength increases with the increase of SiC, the interfacial bonding is good. Large fracture planes gradually transition to small and messy fracture planes, which indicates that SiC particles can refine the grains of 6061 aluminum alloy matrix to increase the strength of the composite.

**Figure 13:**
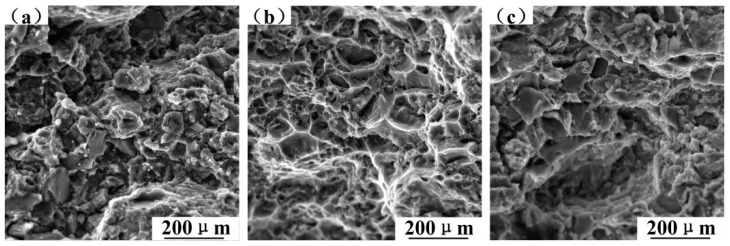
The fracture morphology for tensile temperature: (**a**) T6-treated 30%SiC/6061 at room temperature, (**b**) T6-treated 30%SiC/6061 at 100 °C, and (**c**) T6-treated 30%SiC/6061 at 200 °C.

The fracture morphology of SiC_p_/6061Al composites tested at different temperatures is shown in Figure 11. It was found that the fracture of the composite is brittle at room-temperature because no dimples appear on the fracture morphology, as seen in Figure 13a. The fracture morphology of T6-treated composites at 100 °C, as seen in Figure 13b, has the most obvious tearing ridges, which implies that the elongation of composites at 100 °C is better than others. The softening effect of the matrix increases because of the increase of temperature, which is advantageous for improving the ductility of composites, so that the composite has the maximum elongation at 100 °C. The fracture morphology is characterized by large pits and heaves when the temperature is 200 °C, as shown in Figure 13c, which indicates a brittle fracture feature and a reduced elongation. Li et al. pointed out that the void nucleation in the matrix will be more extensive when the temperature increases, and cracks will initiate in, and propagate along with, the matrix [22]. Excessive softening of the matrix dominates the fracture and most particles simply separate from the matrix along with the interface. These all limit further increases in ductility of the composite with temperature increases.

## 4. Conclusions

In the present paper, the effects of SiC contents and T6-treated on the mechanical properties of SiC_p_/6061Al composites have been studied by carrying out tensile tests at temperatures from room temperature to 200 °C. The main conclusions are briefly summarized as follows:

(1) SiC_p_/6061Al composites, reinforced with mass fractions of SiC particles from 0% to 30%, were successfully prepared by hot press sintering. Optical micrographs show that the SiC particles are homogeneously distributed in the matrix and no pore is found in the composites. The formula of the biggest critical reinforcement concentration of the saturated distribution is proposed to determine the critical value for reinforcement.
(2) The density of composites improves with the increase of SiC content. The high relative density of composites is achieved. The relative density decreases with the increase of SiC content, especially when the mass fraction of SiC reaches 30%.
(3) The effect of T6-treated on the mechanical properties of composites is a marked increase in tensile strength and an obvious decrease in elongation.
(4) With the increase of SiC content, the tensile strength of the composite has been enhanced and the elongation has been reduced. The elongation of the T6-treated composite has insignificant changes with the differences in SiC content. When the mass fraction of SiC particles reaches 30%, both of the elongation and tensile strength of the composite tend to decrease.
(5) The tensile strength of T6-treated composites decreases as temperature increases. The changing of elongation is also not obvious, and the composite has the maximum elongation at 100 °C. The using of T6-treated SiC_p_/6061Al composites needs to be concerned with the strength in the service environment rather than the elongation.

## Figures and Tables

**Table 1:** The chemical composition of 6061 aluminum alloy (wt.%).

Cu	Mn	Mg	Zn	Cr	Ti	Si	Fe	Al
0.15–0.4	0.15	0.8–1.2	0.25	0.04–0.35	0.15	0.4–0.8	0.7	Bal.
